# The Effect of Bariatric Surgery on Quality of Life and Depression

**DOI:** 10.1016/j.jaacop.2025.02.001

**Published:** 2025-02-11

**Authors:** Fien de Boom, Chantal Nederkoorn, Yvonne G.M. Roebroek, Givan F. Paulus, Bjorn Winkens, Nicole D. Bouvy, Ernest van Heurn

**Affiliations:** a Maastricht University Medical Center, Maastricht, the Netherlands; b Maastricht University, Maastricht, the Netherlands; c Amsterdam University Medical Centers, Amsterdam, the Netherlands

**Keywords:** quality of life, depression, bariatric surgery, adolescents, randomized controlled trial

## Abstract

**Objective:**

To assess the effect of bariatric surgery (BS) on health-related quality of life (HRQoL) and depressive symptoms in adolescents in a randomized controlled trial, comparing laparascopic adjustable gastric banding (LAGB) with multidisciplinary lifestyle intervention (MLI) to MLI alone.

**Method:**

Adolescents with severe obesity were referred to our study after failure of conservative therapy and randomized into the intervention group (LAGB and MLI) or the control group (MLI). Anthropometric measurements, questionnaires evaluating HRQoL, and depressive symptoms were taken at baseline, 12 months, and 24 months.

**Results:**

Significant differences between the study groups in body mass index (BMI), HRQoL, and depressive symptoms at 12 months were observed. After 24 months, this difference persisted for BMI (mean difference −3.43, 95% CI −6.78, −0.80) and BMI z score (−0.45, 95% CI −0.75, −0.14) but not for HRQoL (7.80, 95% CI −1.93, 17.53) and depressive symptoms (−4.18, 95% CI −11.68, 3.31).

**Conclusion:**

BS combined with MLI was superior in comparison to MLI alone to reduce BMI and to mediate improvement in HRQoL and self-reported depression after 12 months. However, only the difference in BMI was sustained after 24 months. This indicates that LAGB in combination with MLI does not show superiority over MLI alone for the improvement in HRQoL and depressive symptoms in adolescents with severe obesity over 2 years.

**Clinical Trial Registration Information:**

Bariatric surgery versus conservative treatment in morbidly obese children; https://onderzoekmetmensen.nl/en/node/47066/pdf

## Plain language summary

Bariatric surgery is increasingly performed in adolescents with severe obesity. In our randomized controlled trial, the authors compared the effects of two treatment approaches on health-related quality of life and depressive symptoms: bariatric surgery using laparoscopic adjustable gastric banding combined with multidisciplinary lifestyle intervention versus multidisciplinary lifestyle intervention alone. Over a two-year period, the authors found that laparoscopic adjustable gastric banding combined with multidisciplinary lifestyle intervention did not show superiority over multidisciplinary lifestyle intervention alone for the improvement of health-related quality of life and depressive symptoms.

Obesity is a major public health challenge, increasingly affecting people already from adolescence. It is associated with numerous comorbidities, including psychosocial impairment.^1^, ^2^, ^3^ Psychosocial impairment is highly prevalent among adolescents with severe obesity, often manifesting as a decreased health-related quality of life (HRQoL) and as depression.^4^, ^5^, ^6^ This is associated with weight-related bullying, low self-image, or bodily pain.^7^, ^8^, ^9^ Adolescents referred for bariatric surgery (BS) are vulnerable in terms of psychosocial health. This emphasizes the importance of monitoring mental health during obesity treatment.^10^

Weight loss shows favorable changes in HRQoL and depressive symptoms in adult studies.^11^ Weight loss can be achieved by multiple therapeutic strategies, such as multidisciplinary lifestyle intervention (MLI), medication, and bariatric surgery (BS). The latter has been proved to be an effective method for the remission of multiple obesity-related comorbidities.^12^, ^13^, ^14^, ^15^, ^16^ A systematic review and an original article investigating HRQoL and depression in adolescents after BS found significant improvements in the majority of the included studies.^17^^,^^18^ Two randomized controlled trials (RCTs) investigated HRQoL and depression after BS in this vulnerable group. One recent study by Järvholm *et al.*^14^ showed that after 1 year, mental health aspects improved in the surgical group (the majority of participants underwent Roux-en-Y bypass), whereas they worsened or remained the same in the control group. Nevertheless, after 2 years, mental health was similar among the groups. The RCT by O’Brien *et al.*^15^ evaluated weight loss and HRQoL during 2 years after laparoscopic adjustable gastric banding (LAGB) and observed improved HRQoL in the surgical group compared to the control group. This study, however, did not measure depression, leaving the question of the evolution of depression after LAGB unanswered.

It can be hypothesized that the short-term rapid weight loss that is expected after BS causes improved HRQoL and ameliorates depression because of improved self-image and less physical discomfort. However, when weight loss stagnates or weight (re)gain occurs, psychosocial and mental well-being decrease equally. It is important to gain clarity on this topic, so clinicians can inform their patients about the psychosocial consequences that may occur after BS. Therefore, we investigated the effect of BS on HRQoL and self-reported depression, after 12 and 24 months postintervention (LAGB), using data from the Bariatric Surgery In Children (BASIC) trial.

## Method

### Study Design and Participants

This national study included adolescents with severe obesity between 14 and 16 years of age. Patients who met the inclusion criteria could be referred to our study by a pediatrician or a general practitioner. The surgery and follow-up assessment took place at Maastricht University Medical Centre. The study protocol and 1-year weight loss and glycemic results have been published before.^19^^,^^20^ In summary, inclusion took place between 2011 and 2019, and adolescents with severe obesity (body mass index [BMI] ≥40 or BMI ≥35 with the presence of obesity-related comorbidity) eligible for BS were enrolled in the study. Before inclusion, all patients participated in a lifestyle intervention program for a minimum of 12 months but did not achieve the desired effect (<5% sex- and age-adjusted weight loss). Patients were excluded if psychological contraindications were present, such as eating disorders (binge eating and grazing) and psychopathology (depressive disorder, as diagnosed according to the *DSM-IV* or *DSM-V*).^21^ All participants were assessed by a specialized youth psychologist and dietician. Other exclusion criteria were disorders causing obesity (Prader**–**Willi syndrome, hypothyroidism, prolactinoma), severe cardiorespiratory impairment, insufficient proficiency in the Dutch language, and unwillingness to adhere to the follow-up program. Informed consent was obtained by all participants and their guardians. Ethical approval was obtained from the medical ethics committee of Maastricht University.

### Randomization and Procedure

As previously described by Roebroek *et al.*,^20^ a total sample of 60 participants were targeted to detect meaningful differences between the groups for the primary outcome measure weight loss, while accounting for a 20% loss to follow-up rate. To achieve statistical power of 90% and to detect a 10% difference in total body weight changes (primary outcome) between the study groups (the SD was assumed to be 10% within each group), a total of 22 participants per study group was calculated. The patients were randomized (1:1). The treatment arms consisted of patients receiving LAGB and MLI (designated as the intervention group) or participants undergoing MLI alone (referred to as the control group). The bariatric intervention consisted of LAGB. The MLI consisted of consultations with a dietitian, psychologist, and physical activity, following the Dutch national protocol for MLI in children with obesity.^22^

### Measurements

Medical examination in the hospital was performed at baseline, 12, and 24 months. Examination included measurements of height (cm) and weight (kg) to determine BMI. The BMI *z* score was calculated using sex- and age-specific reference values with the modified LMS (Lambda-Mu-Sigma) method.^23^ The participants were asked to complete psychological questionnaires at the time of each measurement, 2 of which were analyzed for this study. The total score was reported as missing data for unfinished questionnaires, whereas the fully completed subdomains were used for analysis. The primary outcome was change in BMI, and the secondary outcomes were change in HRQoL and depressive symptoms after 12 and 24 months.

#### Pediatric Quality of Life Inventory

HRQoL was obtained by a Dutch translation of the Pediatric Quality of Life Inventory (PedsQL) version 4.0 for teens (13-18 years of age).^24^ This questionnaire comprises 4 subscales: physical functioning (8 items), emotional functioning (5 items), social functioning (5 items), and school functioning (5 items). Scores were calculated as total mean score, physical health summary score, and the mean score of the domains “emotional,” “social,” and “school.” The questionnaire was reverse-scored and linearly transformed to a scale of 0 to 100. A higher PedsQL score indicated better HRQoL. To reverse the score, we then transformed the scale of 0 to 100 as follows: 0 (never a problem) = 100, 1 (almost never a problem) = 75, 2 (sometimes a problem) = 50, 3 (often a problem) = 25, and 4 (almost always a problem) = 0. The following cut-off values were applied: total PedsQL ≤78; PedsQL physical ≤88; PedsQL emotional ≤75; PedsQL social ≤80; Peds-QL school ≤70.^25^

#### Beck Depression Inventory—2

To assess depression in the participants, the Beck Depression Inventory—2 (BDI-2) was used. This questionnaire is composed of 21 multiple-choice questions, answered on a 4-point scale. The questionnaire includes 3 subscales, namely affective (5 items), somatic (9 items), and cognitive (7 items). The questions assess the symptoms and attitudes commonly found in individuals with depression, such as suicidal thoughts, fatigue, and feelings of guilt over the previous 2 weeks. Scores of 0 to 13 indicate minimal depression, 14 to 19 mild depression, 20 to 28 moderate depression, and 29 to 63 severe depression.^26^ A total score of ≥14 was used as indicator for probable despression.^27^ In addition, we report the number of patients who had a total score ≥17, as was used in a previous study in adolescents eligible for BS.^6^ We reported suicidal ideation according to the BDI-2 (item 9 scored as 1, 2, or 3). Suicidal ideation was categorized as: no suicidal ideation (if item 9 was scored as 0), passive suicidal ideation (if item 9 was scored as 1), and active suicidal ideation (if item 9 was scored as 2 or 3).

### Statistical Analysis

Descriptive statistics were computed to provide a summary of the baseline characteristics of the study group. Mean and SD values were calculated for continuous variables, whereas frequencies and percentages were reported for categorical variables. To examine changes in BMI, HRQoL, and self-reported depression over time while accounting for individual variability, marginal models for repeated measures were used. Time (0, 12, 24), group, and the interaction of time by group were included in the fixed part of the model, where an unstructured covariance structure was used to account for the correlation between the repeated measures per subject. The differences in categorical variables between groups were compared using χ^2^, Fisher exact, or Fisher**–**Freeman**–**Halton exact tests. For the exact tests, the 2-sided *p* value was computed as 2 times 1-sided *p* value. All analyses were executed using IBM SPSS for Windows version 27.0. Two-sided *p* values ≤.05 were considered statistically significant.

## Results

### Participants

**Figure 1:**
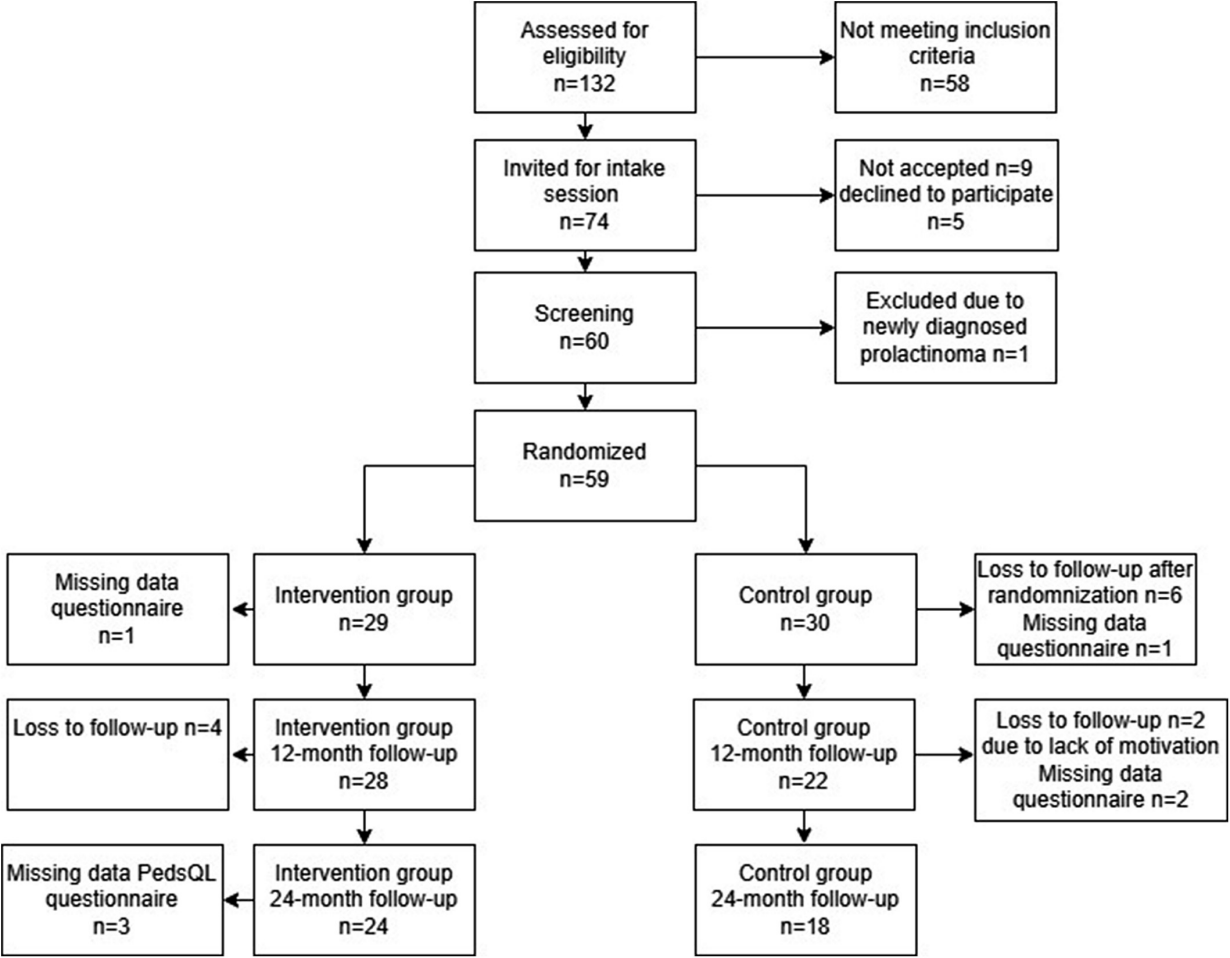
Flow Chart of the Participants in the Study and Follow-Up ***Note:****PedsQL= Pediatric Quality of Life Inventory.*

In total, 59 patients were included in the trial. Table 1 provides an overview of the baseline characteristics of the participants. The mean ± SD age was 15.76 years ± 1.00 year. The majority of participants were female (80%). The participants had a mean body mass index (BMI) of 44.11 (±5.56) and a BMI *z* score of 3.55 (±0.30); the PedsQL total score was on average 69.39 (±13.31), and the BDI-2 score 10.41 (±9.58). Figure 1 is a flow diagram of the participant enrollment and follow-up.

**Table 1:** Baseline Characteristics of the Patients Included in the Analysis (N = 59)

Characteristics	Total N = 59	Control (MLI) n = 30	Intervention (MLI+surgery) n = 29
Age, y	15.76 (±1.00)	15.63 (±0.98)	15.88 (±1.01)
Gender, % female	47 (80%)	24 (80%)	23 (79%)
BMI, kg/m^2^	44.11 (±5.56)	44.29 (±5.59)	43.92 (±5.61)
BMI *z* score^a^	3.55 (±0.30)	3.51 (±0.31)	3.60 (±0.31)
Quality of life			
PedsQL total	69.39 (±13.31)	68.76 (±14.08)	70.03 (±12.68)
PedsQL physical health	70.42 (±14.50)	68.16 (±16.19)	72.77 (±12.37)
PedsQL emotional	67.64 (±19.72)	65.03 (±18.37)	70.34 (±20.10)
PedsQL School	67.76 (±17.81)	66.60 (±19.47)	68.97 (±16.17)
PedsQL Social	67.34 (±18.84)	62.93 (±17.93)	71.90 (±18.96)
Beck Depression Inventory—2			
BDI-2 total	10.41 (±9.58)	10.73 (±9.12)	10.07 (±10.19)
BDI-2 affective	1.63 (±2.37)	1.63 (±2.30)	1.62 (±2.47)
BDI-2 somatic	5.24 (±4.43)	5.30 (±4.40)	5.17 (±4.54)
BDI-2 cognitive	3.63 (±3.82)	3.77 (±3.77)	3.48 (±4.30)

Note: Data for continuous variables are presented as mean (± SD), and categorical data are presented as n (%). BDI-2= Beck Depression Inventory—2; BMI= body mass index; MLI = multidisciplinary lifestyle intervention; PedsQL= Pediatric Quality of Life Inventory.

a BMI z score is sex- and age-adjusted body mass index.

### Missing Data

This study had some missing data (Figure 1). The participants who completed follow-up and those who were lost to follow-up had no significant differences in baseline characteristics, including age, BMI *z* score, BMI, PedsQl total, and BDI-2 total (data not shown).

### Primary Outcome: Effect of BS on BMI

Table 2 shows the intervention effect over time, comparing the intervention group to the control group. Participants in the intervention group showed a significant improvement in BMI compared to the control group after 12 months (mean difference intervention vs control −5.29, 95% CI −7.28, −3.30) and BMI *z* score (−0.56, 95% CI −0.75, −0.14). After 24 months, this significant difference persisted for BMI (mean difference −3.43, 95% CI −6.78, −0.08) and BMI *z* score (−0.45 95% CI −0.75, −0.14).

**Table 2:** Mixed Models for Repeated Measurements Intervention Effect Through Time for Body Mass Index, Quality of Life, and Self-Reported Depression

Variable	Group	n	Baseli ne	n	12 mo	Treatment effect (95% CI)	*p*	n	24 mo	Treatment effect (95% CI)	*p*
BMI	Control	30	44.29 (±1.02)	24	44.67 (±1.28)	–5.29 (–7.28, 3.30)	**<.001**	20	42.45 (±1.27)	–3.43 (–6.78, –0.08)	**.05**
	Interventi on	29	43.92 (±1.04)	29	39.01 (±1.25)			24	38.68 (±1.18)		
BMI *z* score^a^	Control	30	3.52 (±0.05)	24	3.58 (±0.36)	–0.43 (–0.59,–0.26)	**<.001**	19	3.56 (±0.12)	–0.45 (–0.75,–0.14)	**.005**
	Interventi on	29	3.60 (±0.06)	29	3.11 (±0.09)			24	3.20 (±0.10)		
PedsQL											
HRQoL total	Control	30	68.77 (±2.45)	22	67.15 (±2.91)	11.95 (3.13, 20.76)	**.009**	18	71.36 (±3.44)	7.80 (–1.93,17.53)	.11
	Interventi on	29	70.04 (±2.39)	28	80.37 (±2.64)			21	80.43 (±3.20)		
HRQoL physical	Control	30	68.16 (±2.64)	22	69.10 (±3.05)	5.14 (–2.78, 13.06)	.20	19	75.67 (±3.20)	1.14 (–8.11, 10.39)	.81
	Interventi on	29	72.77 (±2.68)	29	78.85 (±2.78)			23	81.42 (±2.93)		
HRQoL emotion al	Control	30	65.03 (±3.60)	22	67.57 (±3.97)	9.54 (0.12, 18.96)	**.05**	19	70.59 (±4.85)	−0.56 (12.45, 11.33)	.93
	Interventi on	29	70.35 (±3.66)	29	82.41 (±3.69)			23	75.35 (±4.52)		

Note: Data are presented as mean (± SD) and intervention effect B (95% CI). p Values were statistically significant at *p* ≤.05. Numbers in boldface type indicate statistical significance. BDI-2 = Beck Depression Inventory—2; BMI = body mass index; MLI = multidisciplinary lifestyle intervention; PedsQL= Pediatric Quality of Life Inventory.

a BMI z score is sex- and age-adjusted body mass index.

### Secondary Outcome: Effects of BS on HRQoL and BDI-2

Further analysis (Table 2) indicated significant differences between the groups in HRQoL total (11.95, 95% CI 3.13, 20.76), HRQoL emotional (9.54, 95% CI 0.12, 18.96), and HRQoL school (9.01, 95% CI 0.94, 17.07) after 12 months, but not after 24 months. The bottom half of Table 2 presents a similar trend for self-reported depression. BDI-2 scores significantly improved in the intervention group compared to the control group after 12 months: BDI-2 total (−6.10, 95% CI −10.64, −1.54), BDI-2 affective (−1.39, 95% CI −2.33, −0.45), BDI-2 somatic (−2.49, 95% CI −4.92, −0.06), and BDI-2 cognitive (−2.33, 95% CI −4.31, −0.36), but not after 24 months.

**Figure 2:**
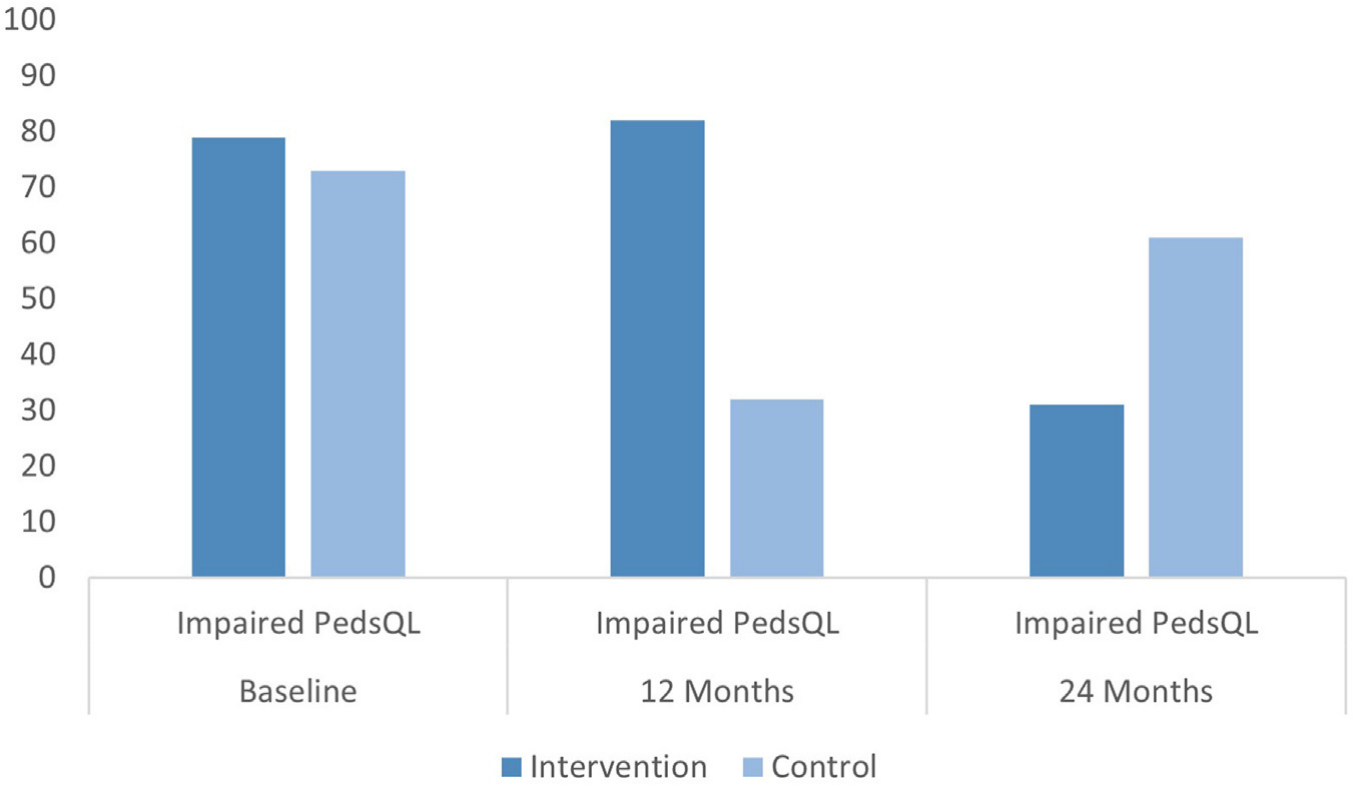
Prevalence of Impaired Health-Related Quality of Life Before and After Surgery During a Two-Year Period ***Note:****Data are presented as proportion of patients (%) with PedsQL total below cut-off value. Cut-off value for impaired HRQoL: PedsQL total ≤78. HRQoL = health-related quality of life; PedsQL= Pediatric Quality of Life Inventory.*

**Figure 3:**
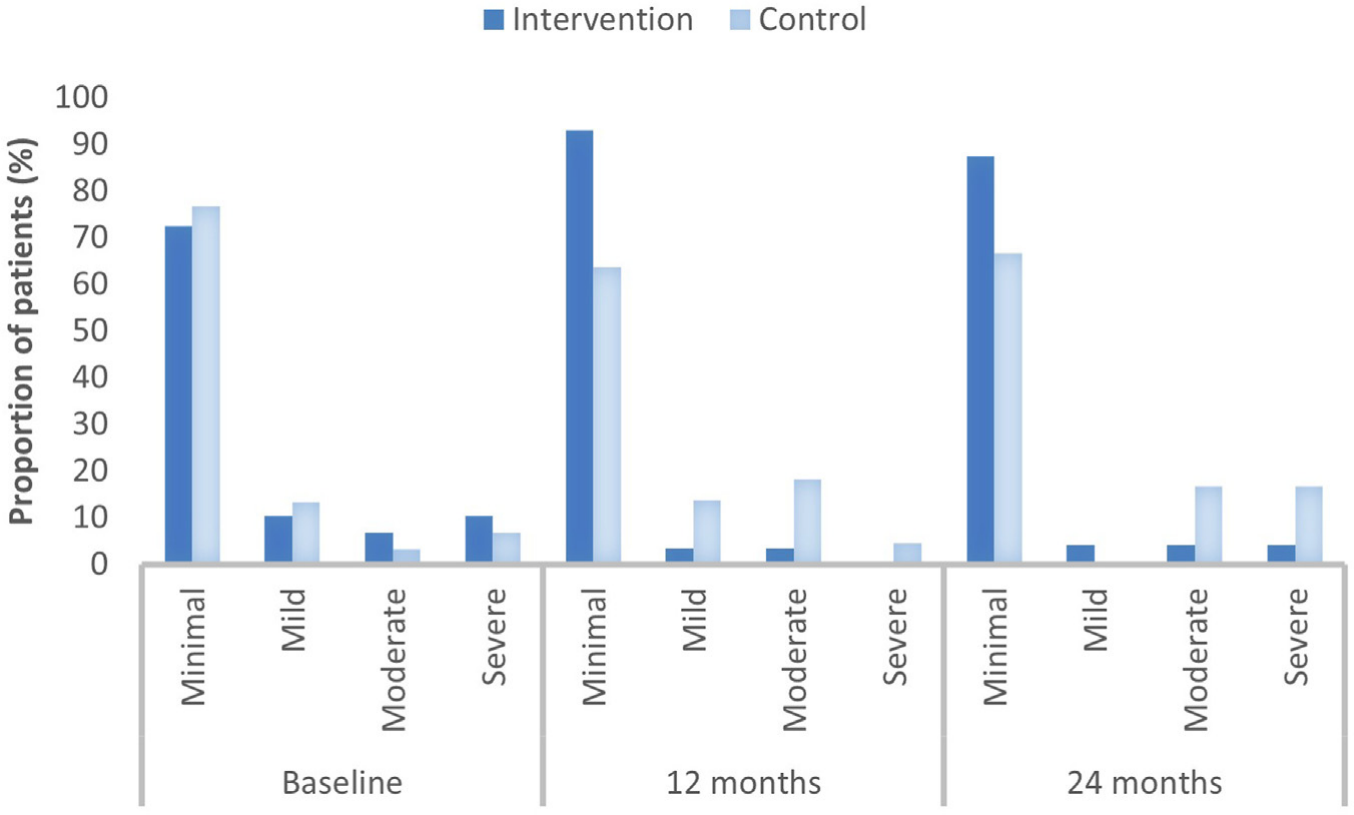
Prevalence of Self-Reported Depression Before and After Surgery During a Two-Year Period ***Note:****Data are presented as proportion of patients (%) with BDI-2 total below cut-off value*. *Scores of 0 to 13 indicate minimal depression, 14 to 19 light depression, 20 to 28 moderate depression, and 29 to 63 severe depression. BDI-2= Beck Depression Inventory—2*.

Figure 2 shows the number of participants with impaired total PedsQL scores; a total of 45 patients (76%) had an impaired PedsQL at baseline. A significantly greater number of participants from the control group had an impaired total PedsQL score (81.8%) compared to the intervention group (32.1%) after 12 months (*p* ≤ .001). After 24 months, 61.1%) of the control group had an impaired HRQoL and 42.9% in the intervention group (*p* = .26). Figure 3 presents the total BDI-2 scores. At baseline, 15 participants (25%) had self-reported depression (BDI-2 total ≥14), and 10 (17%) had a BDI-2 total score of ≥17. After 12 months, the participants in the intervention group scored more frequently minimal on the BDI-2 total scale compared to the control group (93.1% vs 63.6%), while scoring less often for mild (3.4% vs 13.6%), moderate (3.4% vs 18.2%), and severe depression (0% vs 4.5%) (*p* = .07). The same trend was observed at 24 months: minimal (87.5% vs 66.6%), mild (4.2% vs 0%), moderate (4.2% vs 16.6%), and severe (4.2% vs 16.6%) (*p* = .20). Of 59 patients who completed the BDI-2 at baseline, 14 (24%) had some degree of suicidal ideation. Specifically, 13 (22%) scored 1, indicating passive suicidal thoughts, and 1 participant (2%) scored 2, indicating active suicidal ideation. After 12 months, 51 patients completed the BDI-2. In the intervention group, 2 (7%) of the participants had passive suicidal ideation and none of the patients had active ideation. In the control group, 4 (17%) scored passive on the item and 1 participant (4%) scored active. After 24 months, 42 patients completed the BDI-2. In the intervention group, 1 participant (4%) scored passive on the item, and in the control group 4 (22%) scored passive and 1 participant (5%) reported active suicidal ideation.

## Discussion

In this randomized controlled trial in adolescents with severe obesity, BS combined with MLI leading to weight loss showed superior efficacy in comparison to MLI alone for improvement in HRQoL and self-reported depression after 12 months. Despite the sustained difference in BMI (*z* score) after 24 months, the difference in HRQoL and self-reported depression between the study groups did not persist after 24 months.

The prevalence of impaired HRQoL was 76% in this cohort of adolescents with severe obesity. This concerningly high number is likely attributable to the extreme obesity in this study group. It has been observed before that adolescents with the most severe grade of obesity experience the greatest impact on HRQoL.^28^^,^^29^ Depression and obesity are frequently found together, but it remains uncertain whether depression leads to weight gain or vice versa.^30^ In our cohort, 25% of the participants presented with some degree of self-reported depression in which most symptoms were either mild or moderate. One participant was classified with severe depressive symptoms at baseline according to the BDI-2 severity classification. A systematic review^31^ assessing the prevalence of depression in adolescents eligible for BS found depression rates between 15% and 70%. However, comparison with the studies included in this systematic review is complicated because of methodological heterogeneity, namely, the use of different measurement tools and cut-off values for depression. Nonetheless, the high number of patients with impaired HRQoL and depressive symptoms emphasizes the necessity of clinical attention to psychological factors prior to surgery.

Comparing the group that underwent MLI (control) to the group that received MLI and LAGB (intervention group), HRQoL improved more in the intervention group after 12 months. This is in line with previous studies in adults and adolescents.^29^^,^^32^ Nevertheless, the difference between the groups in HrQoL was not sustained after 24 months, whereas weight loss was significantly greater in the intervention group compared to the control group. This suggests that improvements in HRQoL may not be primarily driven by weight loss. The only RCT on this topic, by Järvholm *et al.*,^14^ found comparable results, revealing no significant mental health differences between the surgical (alteration of the digestive tract to both restrict food intake and reduce nutrient absorption [RYGB]) and lifestyle group after 2 years, even though their cohort achieved more weight loss than the participants in the current study (−12.6 kg/m^2^ vs −7.54 kg/m^2^). LAGB, RYGB, and sleeve gastrectomy (SG) differ in their impact on weight loss and eating behaviors. RYGB and SG are more invasive and involve altering the digestive tract to both restrict food intake and reduce nutrient absorption (RYGB) or to reduce stomach size (SG). RYGB and SG lead to more substantial weight loss than does LAGB.^33^ LAGB is a purely restrictive method, which limits food intake and affects eating behaviors. Previous studies found evidence that patients who received LAGB had lower quality of eating compared to patients who underwent RYGB or SG.^34^^,^^35^ This is mainly due to potential discomfort during a meal, struggle with hunger, and the significant changes in eating habits that are needed. Both the lower quality of eating and the difficulty in achieving weight loss can lead to disappointment, affecting HRQoL after LAGB. Despite the varying impact of LAGB and RYGB on weight loss, the RCT by Järvholm *et al.*^14^ and our study indicate that the difference in improvement in HRQoL and mental health between the intervention and the control group is not sustained. The stagnation of HRQoL after the first 12 months may be explained through the reward system. Most of the rapid weight reduction occurs in the first year after BS and is rewarding for patients (“honeymoon phase”). After the first year, this reduction stagnates, necessitating more behavioral and psychological modifications for continued weight loss. Beyond these modifications, factors such as decreased feeling of reward, body dissatisfaction due to excessive skin, and unrealistic expectations regarding rapid weight loss can contribute to stress and create vulnerability for impaired HRQoL. Importantly, HRQoL increased in the control group after the first year and was comparable to that in the intervention group after 24 months. Thus, additional BS does not show superiority over MLI alone for HRQoL improvement.

Self-reported depression decreased more extensively in the intervention group than in the control group in the first 12 months. Nevertheless, after 24 months, this gap between groups became smaller and was no longer statistically and clinically significant. On a group level, BDI-2 score was low, clinically implying that depressive symptoms are minimally present. This is likely attributable to the psychological screening before the study; patients with evident depression assessed by a psychologist during screening were excluded from the trial. Although depressive symptoms decreased in the first 12 months after BS in both adolescents and adult studies, long-term follow-up studies available in adults show high reoccurrence rates of depressive symptoms past the first year mark.^36^ Moreover, the cohort study by Bruze *et al.*^5^ that assessed mental health before and after BS found increased psychiatric health care visits after BS in adolescents. This could be explained by improved accessibility to this care after surgery, but a more plausible explanation is that weight loss does not improve depressive symptoms. Suicidal ideation was relatively low in the intervention group compared to the control group, with only 1 patient scoring “passive suicidal ideation” on the item in the intervention group after 24 months. In line with a previous study in adolescents,^36^ we did not observe increased suicidal ideation in the intervention group.

The main strengths of this study are the randomized design and the fact that no cross-over took place during the study. Although the study was not powered for HRQoL or depression outcomes, we found significant results. Nonetheless, several limitations should be acknowledged. Psychological health was evaluated through questionnaires and a consultation with a specialized psychologist during the screening period. When required, patients received additional support during the follow-up period. A more comprehensive evaluation of mental health during follow-up would have provided a deeper understanding of participants’ mental health. Furthermore, LAGB is not a commonly used BS procedure now. However, at the time of the start of this study, LAGB was frequently used in adults. The main idea behind the use of LAGB was the relatively noninvasive nature and reversibility of the operation in case of long-term band intolerance or failure in adolescents. This study had some loss to follow-up and missing data, although a sensitivity analysis comparing the lost to follow-up group to the group with complete data did not show a significant difference in any baseline demographic or psychosocial feature, suggesting that the loss to follow-up might be completely at random.

To conclude, the findings of this study suggest that LAGB combined with MLI (intervention group) and MLI alone (control group) had similar outcomes in psychosocial health after 2 years, although weight loss was significantly greater in the intervention group. We underscore the importance of monitoring psychosocial health before and after BS in this vulnerable group, as it allows for quick access to specialized support for adolescents who have undergone BS. Long-term longitudinal studies powered for psychosocial outcomes are needed to further assess the impact of BS on psychosocial health in adolescents, as it may fluctuate over time.

## CRediT authorship contribution statement

**Fien de Boom:** Writing – review & editing, Writing – original draft, Formal analysis, Conceptualization. **Chantal Nederkoorn:** Writing – review & editing, Supervision, Methodology, Investigation. **Yvonne G.M. Roebroek:** Writing – review & editing, Methodology, Investigation, Data curation. **Givan F. Paulus:** Writing – review & editing, Methodology, Investigation, Data curation, Conceptualization. **Bjorn Winkens:** Writing – review & editing, Methodology, Formal analysis, Data curation. **Nicole D. Bouvy:** Writing – review & editing, Supervision, Methodology, Conceptualization. **Ernest van Heurn:** Writing – review & editing, Supervision, Project administration, Methodology, Funding acquisition, Conceptualization.
